# mGluR5, CB1 and neuroprotection

**DOI:** 10.18632/oncotarget.14086

**Published:** 2016-12-21

**Authors:** Toniana G. Carvalho, Juliana G. Doria, Fabiola M. Ribeiro

**Affiliations:** Departament of Biochemistry and Immunology, Institute of Biological Science, Universidade Federal de Minas Gerais, Belo Horizonte, Brazil

**Keywords:** mGluR5, CB1, neuroprotectionh, AKT, ERK1/2, Neuroscience

The metabotropic glutamate receptor 5 (mGluR5) is a Gα_q/11_-coupled receptor, mainly found at the postsynaptic site. mGluR5 stimulation leads to the activation of phospholipase Cβ1 (PLCβ), promoting diacylglicerol (DAG) and inositol 1,4,5-trisphosphate (IP_3_) formation, which leads to the release of Ca^2+^ from the intracellular stores and the activation of protein kinases, including protein kinase C. Additionally, stimulation of mGluR5 also triggers the activation of other cell signaling pathways that are important for cell proliferation and survival, such as the activation of the extracellular signal regulated protein kinase (ERK) and AKT. Recently, we have demonstrated that the mGluR5 positive allosteric modulator (PAM), CDPPB, activates AKT without increasing intracellular Ca^2+^ and protects neurons from glutamate-induced neuronal cell death [1]. Furthermore, we have shown that CDPPB treatment prevents the cognitive deficit and diminishes neuronal cell loss and huntingtin aggregate formation in a mouse model of Huntington’s disease, the BACHD mice [2]. In addition, mGluR5 has also been implicated in the pathology of other neurodegenerative diseases, including Alzheimer and Parkinson’s disease, as well as in physiological processes, including memory and motor coordination [3].

The cannabinoid receptor 1 (CB1) is a Gα_i/o_-coupled receptor mainly present at the presynaptic terminals and that can be activated by synthetic, plant-derived and endogenous cannabinoids, including anandamide and 2-arachidonoylglycerol (2-AG). CB1 has been implicated in several biological and pathological processes, playing a role in the regulation of cell proliferation, apoptosis and metastasis formation [4]. CB1 activation can limit the release of neurotransmitters, such as gamma-aminobutyric acid (GABA) and glutamate, from the presynaptic site, as well as activate neuroprotective pathways, decreasing excitotoxicity [5].

More recently, we have demonstrated that mGluR5 and CB1 can act together to promote neuroprotection [6]. Using primary cultured corticostriatal neurons, we have shown that CDPPB-mediated neuroprotection against glutamate insult can be reversed by both MPEP, which is a mGluR5 blocker, and AM251, a CB1 antagonist. Moreover, the neuroprotection induced by URB597 and JZL184, which are inhibitors of anandamide and 2-AG degradation, respectively, can also be abrogated by MPEP and AM251. Corroborating these data, CDPPB, URB597 and JZL184 were unable to promote neuroprotection of either mGluR5^−/−^ or CB1 knockdown neurons. In addition, neuroprotection by CDPPB, URB597 and JZL184 was dependent on the activation of pathways that lead to the stimulation of AKT and ERK1/2, but was independent of alterations in intracellular Ca^2+^ concentration or glutamate release.

In several neurodegenerative diseases, neuronal cell death is preceded by synaptic loss, which is usually responsible for the early cognitive deficits. The study by Batista et al, 2016 [6], showed that CDPPB protected the postsynaptic site and that this protection was blocked by MPEP and AM251. In addition, JZL184 protected the presynaptic terminals and, to a lesser extent, the postsynaptic site. Curiously, AM251 reversed JZL-mediated protection of both the pre and postsynapse, while MPEP only abolished the protection of the postsynaptic site. mGluR5 can regulate the synthesis of 2-AG at the postsynaptic site through the activation of PLC-β [7]. Thus, we hypothesize that JZL184 is not enough to cause postsynaptic protection when mGluR5 is blocked and unable to contribute to 2-Ag synthesis. However, since JZL184 blocks 2-AG degradation by inhibiting the presynaptic enzyme monoacylglycerol lipase (MGL), 2-AG levels will be more likely to be increased at the presynaptic terminal than at the postsynaptic site. Thus, in the presence of MPEP and JZL184, 2-AG levels would still be high enough to promote neuroprotection of presynaptic terminals, but not of postsynaptic sites.

**Figure 1:**
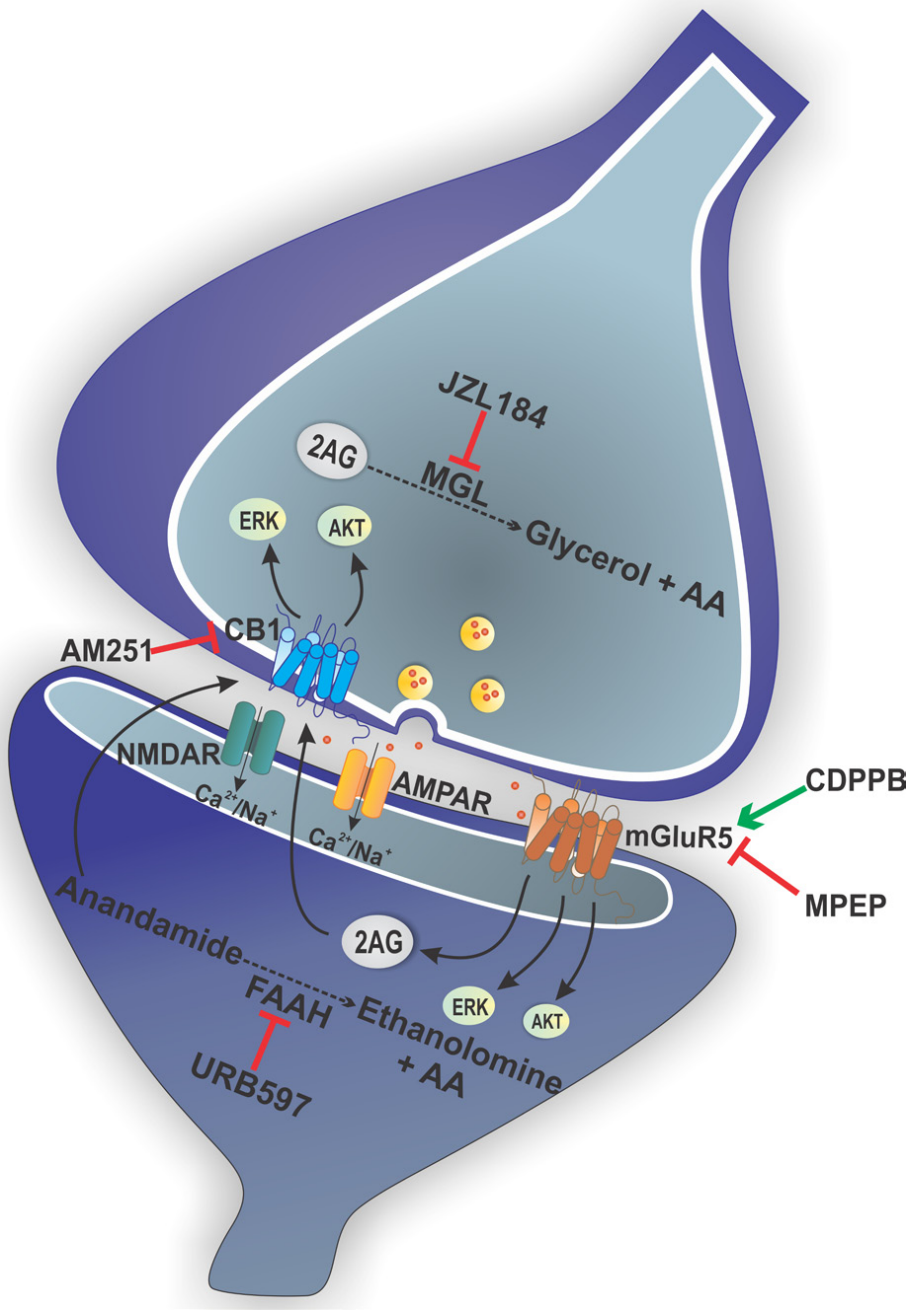
Schematic cartoon displaying summary of the cell signaling pathways activated by mGluR5 and CB1 to promote neuroprotection

From the study published by Batista et al, 2016 [6], as well as by other studies published by our group and others, it is clear that there is a high level of complexity involving the modulation of the cell signaling pathways that regulate cell survival/proliferation via mGluR5/ CB1 activation. Notably, the crosstalk between mGluR5 and CB1 appears to be crucial to promote neuronal survival. However, these receptors can activate a wide variety of cell signaling pathways and produce several second messengers, making it difficult to understand the mechanism underlying neuroprotection and preservation of post and presynaptic sites. Therefore, a better understanding of the crosstalk between mGluR5 and CB1 and the investigation of the cell signaling pathways stimulated by these receptors will be important for the development of future drugs to treat neurodegenerative diseases. So far, no disease-modifying drug has been developed to treat neurodegenerative diseases. It is possible that the complexity of the cell signaling pathways that are activated by these receptors make it difficult for agonists and antagonist to become effective drugs to treat CNS disorders. In this sense, mGluR5 PAMs are very promising drugs, as these modulators are bias agonists and can activate one cell signaling pathway without triggering others [1, 2]. In addition, JZL184 and URB597 are not direct CB1 agonists, but only increase the endogenous levels of cannabinoids, thus having a low chance of causing harmful health effects. Therefore, CDPPB, JZL184 and URB597 are promising drugs to treat neurodegenerative diseases, exhibiting a potent neuroprotective effect and having the potential to exhibit low levels of adverse effects.
